# Scientific Items

**Published:** 1881-06-20

**Authors:** 


### SCIENTIFIC ITEMS.

**Trees and Lightning.**—Professor Colladon, of Geneva, published the conclusion several years ago that, when lightning strikes a tree, it is received on the ends of the branches, which, being excellent conductors, lead it, without suffering disturbance, down to the larger limbs. Thence it descends to the main limbs and the trunk, whose conducting power, intrinsically inferior to that of the smaller and younger shoots of the top, is insufficient to sustain the concentrated force of the currents which have united here from the thousand channels by which they have so far descended. Here, then, generally appear the first marks of the shock, not because the lightning has struck the tree at that place, as might be superficially supposed, but because the conducting powers of the tree begin to fail at this point. This view was satisfactorily confirmed by the effect of the lightning upon a poplar tree, which was struck at Geneva on the 5th of May, 1880. The young, tender leaves of the main topmost branch of this tree and of the branches immediately below it were torn up into small fragments, which strewed the ground below them, as if they had undergone a violent shock of air, such as would be produced by an explosion of dynamite. Many trees may be compared, in respect to their power to conduct electricity, to structures of wood or masonary, which are well furnished with conductors on their upper part, but with which no conducting connection with the ground is given. If such a building were struck with lightning, its upper part would not be hurt, while its lower part would suffer badly.

The danger of being struck by lightning, to which persons standing under a tree are exposed, is thus accounted for. The top of the tree, bristling with conducting twigs, attracts the lightning; the current, meeting with non-conducting obstacles at the trunk, jumps from it to the surrounding bodies, whether they be bushes or men and animals. Of two persons, one standing under the tree, the other sitting among the limbs at the top, the latter would be in a vastly safer position. Birds having nests in trees are rarely struck by lightning, and their nests are hardly ever damaged. Large trees growing near a house will protect it from lightning, provided there is no pond or well or stream beyond the house to attract the current across it. If the water is on the same side of the house as the tree, or the tree is between it and the house, or has a rod attached to it, the protection is almost perfect.—*Popular Science Monthly.*

**Refrigeration and Animal Heat.**—Dr. Paul Delmas, of Bordeaux, has published the results of some experiments in refrigerating a healthy person by exposing him, during from a quarter of a minute to five minutes, to a bath of water at 50°, in which he took notice of the temperature of the subject during the exposure and every five minutes in succeeding hours. During the application of the cold, while the subject showed every sign of very intense sensations, the temperature of the body hardly varied at all, or, at most, less than half a degree from that recorded in the beginning. It still varies but little after the application is over, if, having been dried and dressed, the subject remains perfectly still; but if he exert himself actively, either immediately or after a time of immobility, so as to bring on the external phenomena of cold reaction, the temperature suddenly falls. The reduction persists for several hours, and is more pronounced as the sensation of heat in the subject is stronger. On the other hand, if chill continue or reappear, the animal temperature either does not fall or begins to rise again. The pulse suddenly becomes very quick at the beginning of the cold application; its velocity diminishes after a few seconds, and by the end of the experiment returns to the original rate, or falls below it. The retardation stops or progresses slowly if the subject keeps quiet, but becomes more pronounced and persistent as he gives signs of energetic reaction and of a general sensation of heat.—*Pop. Month.*

**Precocity a Sign of Inferiority.**—M. D. Delaunay, in a communication to the French Societe de Biologie, has advanced the opinion that precocity is a sign of biological inferiority. In support of his position, he adduces the fact that the lower species develop more rapidly, and are at the same time more precocious than those higher in the scale. Man is the longest of all in arriving at maturity, and the inferior races of men are more precocious than the superior, as is seen in the children of the Esquimaux, Negroes, Cochin-Chinese, Japanese, Arabs, etc., who are, up to a certain age, more vigorous and more intellectual than small Europeans. Precociousness becomes less and less in proportion to the advance made by any race in civilization—a fact which is illustrated by the lowering of the standard for recruits, which has been made necessary in France twice during the present century, by the decreasing rapidity of growth of the youth of the country. Women are more precocious than men, and in all domestic animals the female is formed sooner than the male. From eight to twelve years of age a girl gains one pound a year on a boy, and in mixed schools girls obtain the first places up to the age of twelve. The inferior tissues and organs develop before the higher ones, and the brain is the slowest of all organs to develop. M. Delaunay concludes his paper by stating that the precocity of organs and organisms is in an inverse ratio to the extent of their evolution.—*Ec. Med. Jour.*

**Telegraphing without Wires.**—Prof. Loomis has been for some months experimenting in the West Virginia mountains on his aerial telegraphy, and has succeeded, by running up wires to a certain altitude, in reaching the current of electricity which he claims can be found at that height, and by means of which communication can be had at any distance. It is said the professor has telegraphed to parties eleven miles distant, by merely sending up a kite at each end of the distance to a certain height, attached to which, in place of an ordinary string, was a fine copper wire. When both kites touch the same current, communication was had between them, and messages were sent from one end to the other by means of the ordinary Morse instrument in connection with the instrument invented by Prof. Loomis. He now has a project for a series of experiments from a point on one of the highest peaks on the Alps, in Switzerland, to a similarly situated place in the Rocky Mountains on this side of the world.—*Com. Bul.*

